# TNF-*α*/NF-*κ*B/Snail pathway in cancer cell migration and invasion

**DOI:** 10.1038/sj.bjc.6605530

**Published:** 2010-01-19

**Authors:** Y Wu, B P Zhou

**Affiliations:** 1 Departments of Molecular and Biomedical Pharmacology, University of Kentucky School of Medicine, Lexington, KY 40506, USA; 2 Departments of Molecular and Cellular Biochemistry, University of Kentucky School of Medicine, Lexington, KY 40506, USA; 3 Markey Cancer Center, University of Kentucky School of Medicine, Lexington, KY 40506, USA

**Keywords:** TNF-*α*, NF-*κ*B, Snail, EMT

## Abstract

Tumour necrosis factor-alpha (TNF-*α*) is an important inflammatory factor that acts as a master switch in establishing an intricate link between inflammation and cancer. A wide variety of evidence has pointed to a critical role of TNF-*α* in tumour proliferation, migration, invasion and angiogenesis. The function of TNF-*α* as a key regulator of the tumour microenvironment is well recognised. We will emphasise the contribution of TNF-*α* and the nuclear factor-*κ*B pathway on tumour cell invasion and metastasis. Understanding the mechanisms underlying inflammation-mediated metastasis will reveal new therapeutic targets for cancer prevention and treatment.

Tumour necrosis factor (TNF)-*α* is a key cytokine involved in inflammation, immunity, cellular homeostasis and tumour progression (Balkwill, 2009). It was first identified as an anti-tumor cytokine accompanied by serious toxicity involving in the innate and adaptive immune system. It is required for proper proliferation and function of NK cells, T cells, B cells, macrophage and dendritic cells and is an important effector molecule in cell-mediated killing of certain tumours. However, emerging evidences have shown TNF-*α* was one of the major mediators of cancer-related inflammation and acted as a tumour-promoting factor. This apparently paradoxical effect of TNF-*α* on tumour may reflect the difference in chronic synthesis and acute high-dose local administration. High doses of human recombinant TNF-*α*-induced haemorrhagic tumour necrosis of both syngeneic and xenografted tumours when injected locally and repeatedly (Balkwill, 2009). By contrast, low dose, chronic TNF-*α* has angiogenic activity and promotes tumour progression.

Tumour necrosis factor-*α* is synthesised as a transmembrane protein with a molecular mass of 26 kDa and the pro-peptide is secreted as a soluble 17-kDa molecule on cleavage by TNF-*α*-converting enzyme (TACE). Although activated macrophages are the major source of TNF-*α*, it can also be produced by a variety of other cells, such as fibroblasts, astrocytes, Kupffer cells, smooth muscle cells, keratinocytes and a wide variety of tumour cells, including B-cell lymphoma, breast and colon carcinomas. Increasing evidences indicate that TNF-*α* acts as tumour-promoting factor and is linked to all steps of tumourigenesis including transformation, proliferation, angiogenesis, invasion and metastasis in many cancers. Tumour necrosis factor-*α* has a particularly important role in tumour microenvironment and promotes tumour cell migration and invasion, however, the mechanism by which TNF-*α* facilitates these events remains elusive. In this study, we discuss the molecular mechanisms of TNF-*α*-induced tumour migration and invasion, particularly focusing on the contribution of TNF-*α*–NF-*κ*B–Snail pathway.

## TNF-*α*/NF-*κ*B Signalling Pathway

**Figure 1:**
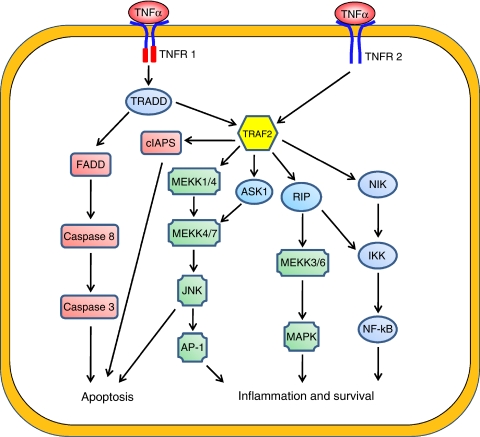
The downstream signalling pathways of TNF-*α*. The TNF-*α* can activate different pathways to induce apoptosis, cell survival or inflammation. Tumour necrosis factor induces the apoptosis by binding caspase-8 to FADD and promotes inflammation and survival, which is mediated through TRAF2 via JNK-dependent kinase cascade, MEKK kinase cascade and NF-*κ*B activation by RIP.

Tumour necrosis factor-*α* secretion can be induced by conserved structural elements common to microbial pathogens, such as lipopolysaccharide (LPS), that are bound by Toll-like receptors (TLRs) (Aderem and Ulevitch, 2000). The TLRs transcriptionally induce proinflammatory cytokines, including TNF-*α*, through the convergence of the nuclear factor (NF)-*κ*B and NF-AT signalling pathways, and thereby enhance translational efficiency by a mechanism targeting consensus 3′-untranslated AU-rich elements (ARE) in mRNA (Dumitru *et al*, 2000). Tumour necrosis factor-*α* mediates its effect through two different receptors: TNF-*α* receptor I (TNF-R1; p55 or p60) and TNF-*α* receptor II (TNF-R2; p75 or p80) (Aggarwal, 2003). The TNF-R1 and TNF-R2 belong to the TNF super family receptors that have structurally related cysteine-rich extracellular domain. The TNF-R2 is expressed only on endothelial and immune cells. Although TNF-R2 has been shown to mediate signals that promote tissue repair and angiogenesis, the functional consequences of TNF-R2 signalling are not well characterised. The TNF-R1 is universally expressed on all cell types and has a broader role in NF-*κ*B activation versus that of TNF-R2. The TNF-R1 ligation induces receptor trimerisation and the recruitment of the adaptor protein TNF-R1-associated death domain protein (TRADD) that binds to a specific death domain (DD) in the cytoplasmic domain of TNF-R1. TNF-R1-associated death domain protein also recruits TNF receptor-associated factor (TRAF2) and activates I*κ*B kinase (IKK) through receptor-interacting protein (RIP). The RIP1 is ubiquitinated in a TRAF2-dependent manner during TNF-R1 activation and is essential for TNF-*α*-induced IKK and NF-*κ*B activation. RIP1 knock-out cells fail to activate IKK in response to TNF-*α*. The TRAF2 recruits the IKK complex to the activated TNF-R1 by interacting with the LZ motifs of IKK*α* and IKK*β*. The IKK complex consists of IKK*α*, IKK*β* and NEMO (also known as IKK*γ*). In the classical activation pathway, activated IKK*β* phosphorylates specific serine residues of I*κ*B in a NEMO-dependent manner, leading to I*κ*B phosphorylation, ubiquitination and proteosome-mediated degradation. The degradation of I*κ*B releases the transcription factors NF-*κ*B, which translocates to the nucleus, binds the *κ*B site and activates gene transcription. Nuclear factor-*κ*B is composed of five distinct but structurally related subunits, p50, p52, c-Rel, RelA and RelB. These subunits can form various homodimeric and heterdimeric complexes; each combination of subunits has a specific signalling function (Ghosh *et al*, 1998). These subunits are transcriptionally inactive when they form complexes with cytoplasmic I*κ*B family proteins. Ligation of TNF-R1 is both necessary and sufficient to induce the cytotoxic and proinflammatory TNF-*α* response. The TNF-R2 may contribute to TNF-R1 responses at low concentrations of TNF-*α*, in which TNF-R2 captures TNF-*α* and passes it to TNF-R1 (Bradley, 2008). Although the TNF-*α* signal transduction pathway is complex (Figure 1) and not fully understood, the pro-inflammatory effects of TNF-*α* are primarily because of its ability to activate NF-*κ*B whereas the anti-tumor effects are due to activation of Caspase 3 and induction of apoptosis. In almost all cell types, when exposed to TNF-*α*, NF-*κ*B is activated and leads to the expression of a variety of inflammation-related genes. Transient activation of NF-*κ*B in response to stimulation by cytokines induces the inflammatory response; however, sustained activation of NF-*κ*B has been associated with several aspects of oncogenesis, such as promoting cancer-cell proliferation, preventing apoptosis in drug resistance and increasing tumour angiogenesis and metastasis.

## TNF-*α* in tumourigenesis

The association of inflammation and cancer has been well recognised in many types of cancer and inflammation has been regarded as the ‘seventh hallmark of cancer’ (Mantovani *et al*, 2008; Mantovani, 2009). Accumulating evidence has shown that TNF-*α* is a key mediator of inflammation and cancer (Sethi *et al*, 2008; Balkwill, 2009). Constitutive production of TNF-*α* from the tumour microenvironment is a characteristic of many malignant tumours and its presence is often associated with poor prognosis. As TNF-*α* receptors are expressed on both epithelial and stromal cells, TNF-*α* can directly facilitates cancer development by regulating the proliferation and survival of neoplastic cells and it can also exert its effects indirectly through endothelial cells and other inflammatory cells presented at the tumour microenvironment. Tumour stromal cells, including macrophages, dendritic cells and fibroblasts, generate several inflammatory cytokines such as TNF-*α*, IL-1 and IL-6. These cytokines attract and recruit more inflammatory cells to the tumour microenvironment to further enhance the proliferation and survival of genetically altered tumour cells. Furthermore, the inflammatory nature of the tumour microenvironment can lead to additional genetic changes in cells associated with malignancy.

Tumour necrosis factor-*α* is involved in all steps of tumourigenesis (Balkwill, 2009). First, TNF-*α* induces tumour initiation and promotion. Either TNF-*α* or TNF-*α* receptors deficient mice have reduced susceptibility to chemically induced skin cancers and develop fewer experimental metastases. In TNF-*α*-deficient mice, okadaic acid has reduced tumour-promoting activity and the development of TPA-induced skin cancer is delayed. Inhibition of TNF-*α* results in a marked reduction in tumour onset and tumour burden (Karin and Greten, 2005).

Tumour necrosis factor-*α*-induced tumour initiation and tumour promotion are mediated by the activation of NF-*κ*B-, PKC*α*- and AP-1-dependent pathways. Nuclear factor-*κ*B is critical for TNF-*α*-induced tumour promotion. In mouse epidermal JB6 cells, TNF-*α* treatment increases NF-*κ*B activity in a dose-dependent manner and TNF-*α*-induced NF-*κ*B activation is essential for neoplastic transformation of these cells (Hsu *et al*, 2001). Second, TNF-*α* enhances tumour cell proliferation. It serves predominantly as a mutagen to promote the proliferation and survival of many tumour cell lines without inducing cell differentiation. Once again, NF-*κ*B activation is essential for TNF-*α*-induced survival and proliferation. Inhibition of nuclear translocation of NF-*κ*B specifically blocks TNF-*α*-induced cell proliferation. The TNF-*α* also promotes tumour cell survival through the induction of genes encoding NF-*κ*B-dependent antiapoptotic molecules (Shishodia and Aggarwal, 2004). In addition, TNF-*α* not only acts as an autocrine growth factor but also induces the expression of other growth factors such as amphiregulin, EGFR and TGF-*α*, leading to increased tumour proliferation. Third, TNF-*α* enhances tumour angiogenesis. It mediates tumour angiogenesis through various angiogenic factors such as IL-8 and VEGF, and also is a critical regulator of VEGF and jagged-1 expression via a JNK- and AP-1-dependent pathway (Johnston *et al*, 2009). Neutralising TNF-*α* function with a polyclonal antibody completely blocks its angiogenic activity (Leibovich *et al*, 1987). Finally, TNF-*α* also confers an invasive and transformed phenotype onto tumour cells. Pre-treatment of the animals with TNF-*α* increases lung metastases in an experimental fibrosarcoma (Orosz *et al*, 1993). However, neutralising endogenous TNF-*α* with an anti-TNF-*α* antibody reduces lung metastasis. Tumour necrosis factor-*α*-mediated signalling maintains tumour neovascularisation partly by inducing hepatocyte growth factor (HGF) to support lung metastasis (Tomita *et al*, 2004). The TNF-*α* also induces tumour cell invasion through NF-*κ*B- and JNK-mediated upregulation of migration-inhibitory factor (MIF) in macrophages and through enhanced MMPs production in tumour cells (Hagemann *et al*, 2005). In addition, in some cancer cells, TNF-*α* enhances cells migration and metastasis through NF-*κ*B-dependent induction of the chemokine receptor CXCR4, monocyte chemoattractant protein-1 (MCP-1), IL-8 and intercellular adhesion molecule-1 (Kulbe *et al*, 2005). The TNF-*α* signalling through NF-*κ*B in resident macrophages creates an inflammatory microenvironment that enhances LLC cells to metastasise (Stathopoulos *et al*, 2008). It can also promote breast cancer cell migration through upregulation of LOX (Liang *et al*, 2007). Furthermore, TNF-*α* enhances the invasiveness of tumour cells through induction of MMPs or *α*2*β*1 integrin (Montesano *et al*, 2005). Importantly, both exogenous and macrophage-produced TNF-*α* accelerate the epithelial-mesenchymal transition (EMT). The TNF-*α* enhances the invasive property of cancer cells by inducing EMT through Snail- or ZEB1/ZEB2-dependent mechanisms (Chua *et al*, 2007; Chuang *et al*, 2008). Therefore, TNF-*α* promotes tumour metastasis through its effects on tumour cells and stromal and inflammatory cells within the tumour microenvironment.

## The network between the TNF-*α*/NF-*κ*B and Snail during EMT

**Figure 2:**
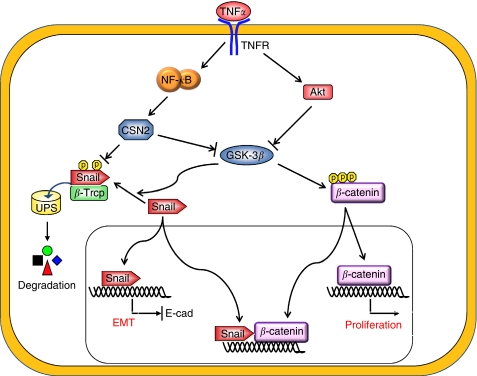
An overview of the signalling pathways mediated by TNF-*α* in metastasis. The TNF-*α* induces protein stabilisation of Snail and *β*-catenin by inhibiting GSK-3*β*-mediated phosphorylation through NF-*κ*B and Akt signalling pathways. It also induces CSN2 expression through a NF-*κ*B-dependent pathway. Together, these signalling events contribute to EMT induction and invasion in tumour cells.

Epithelial-mesenchymal transition is a complex stepwise phenomenon that occurs during embryonic development and tumour progression, and it also has a crucial role in chronic inflammatory and fibrogenic disease (Thiery and Sleeman, 2006). It is characterised by the disruption of intercellular junctions, replacement of apical-basolateral polarity with front-to-back polarity and acquisition of migratory and invasive phenotypes. It is a critical early event for the invasion and metastasis of many carcinomas (Cardiff, 2005; Thompson *et al*, 2005). The loss of E-cadherin is the hallmark of EMT. Several transcription factors have been implicated in the transcriptional repression of E-cadherin, including zinc-finger proteins of the Snail/Slug family, Twist, ZEB1, SIP1, and the basic helix-loop-helix factor E12/E47. Snail was the first discovered and is the most important transcriptional repressor of E-cadherin. Snail was identified in *Drosophila* as a suppressor of the transcription of *shotgun* (an E-cadherin homologue) in the control of embryogenesis (Nieto, 2002; Barrallo-Gimeno and Nieto, 2005). Snail has a central role in morphogenesis, as it is essential for the formation of the mesoderm and neural crest, which requires large-scale cell movements in organisms ranging from flies to mammals. Absence of Snail is lethal because of severe defects at the gastrula stage during development (Carver *et al*, 2001). Snail has a fundamental role in EMT and breast cancer metastasis by suppressing E-cadherin expression. In fact, overexpression of Snail was recently found in both epithelial and endothelial cells of invasive breast cancer but was undetectable in normal breast (Parker *et al*, 2004; Martin *et al*, 2005). The expression of Snail in breast carcinomas is associated with metastasis, tumour recurrence and poor prognosis (Peinado *et al*, 2007). Snail also downregulates the expression of other epithelial molecules, including Claudins, Occludins and Muc1 and induces the expression of genes associated with a mesenchymal and invasive phenotype, such as fibronectin and MMP9. Expression of Snail is regulated by a complex integrated signalling network; this includes integrin-linked kinase (ILK), phosphatidylinositol 3-kinase (PI3-K), mitogen-activated protein kinases (MAPKs), glycogen synthase kinase 3-beta (GSK-3*β*) and NF-*κ*B pathways (De Craene *et al*, 2005). Snail expression is regulated by the NF-*κ*B pathway through transcriptional and post-translational mechanisms. First, Snail expression is directly activated by the NF-*κ*B homologue, Dorsal, in drosophila (Ip *et al*, 1992). Nuclear factor-*κ*B also binds the human snail promoter between −194 and −78 bp, leading to increased Snail transcription (Barbera *et al*, 2004). Recently, Raf kinase inhibitor protein (RKIP), a metastatic suppressor, was shown to inhibit NF-*κ*B activity, and conversely, Snail can repress the expression of RKIP. Therefore, there is a circuitry between RKIP, NF-*κ*B and Snail, in which overexpression of Snail in tumours inhibits RKIP and induce EMT (Katsman *et al*, 2009; Wu and Bonavida, 2009). In addition, GSK-3*β* inhibition stimulates the transcription of Snail by activating the NF-*κ*B pathway (Bachelder *et al*, 2005). Furthermore, in human mammary epithelial MCF-10A cells, overexpressing a constitutively active Type I insulin-like growth factor receptor (IGF-1R) leads to the activation of Akt, suppression of GSK-3*β* and activation of NF-*κ*B. This results in increased Snail expression, downregulation of E-cadherin and the subsequent induction of EMT (Kim *et al*, 2007). Tumour necrosis factor-*α* can also activate Akt, which stimulates NF-*κ*B by directly phosphorylating IKK*α*, and this results in the upregulation of Snail and induction of EMT (Julien *et al*, 2007). Previously, we have shown that Snail is a highly unstable protein targeted for degradation by GSK-3*β*-dependent phosphorylation and SCF^*β*−TRCP^-mediated ubiquitination (Zhou *et al*, 2004). In our recent study, we found that TNF-*α* is the major signal that induces Snail stabilisation and EMT induction (Wu *et al*, 2009). We showed that TNF-*α* greatly enhanced the migration and invasion of tumour cells by inducing the EMT programme through NF-*κ*B-mediated Snail stabilisation. Knockdown of Snail expression not only inhibits TNF-*α*-induced cancer cell migration and invasion *in vitro* but also suppresses LPS-mediated metastasis *in vivo*. The TNF-*α*/NF-*κ*B-stabilised Snail is mediated by the transcription induction of CSN2, which inhibits the phosphorylation and ubiquitination of Snail by disrupting the binding of Snail to GSK-3*β* and *β*-Trcp, and results in the stabilisation of Snail in a non-phosphorylated and non-ubiquitinated functional state (Figure 2). CSN2 is the second and most conserved component of the eight subunits of COP9 signalosome (CSN) complex, which controls the functional assembly and activity of cullin-RING ubiquitin ligases (CRLs). The majority of protein degradation in cells occurs through the ubiquitin-mediated proteolytic pathway that catalyses the covalent attachment of ubiquitin to target proteins by the concerted actions of three enzymes, E1 (ubiquitin-activating enzyme), E2 (ubiquitin-conjugating enzyme) and E3 (ubiquitin ligase). Ubiquitin E3 ligases provide the substrate specificity for ubiquitination reaction. Cullin-RING ubiquitin ligase forms multisubunit complexes including a cullin, a RING H2 finger protein (Rbx1 or 2), an adaptor subunit (e.g., Skp1 for Cul1), and an F-box protein (Nalepa *et al*, 2006). Cullin is a scaffold protein that serves as the assembly centre for the recognition components of a large variety of ubiquitin E3 ligases and their cognate ubiquitin E2 enzymes. For example, the C-terminus of Cul1 binds Rbx1 that facilitates the recruitment of the E2 to the complex, whereas the N-terminus of Cul1 associates the adaptor Skp1, which links to an F-box protein through the F-box motif. The F-box protein binds and positions the substrate for ubiquitination by the E2. There are seven Cullins in human and all cullins contain a conserved lysine residue in its C-terminus that can be conjugated to the ubiquitin-related protein Nedd8 (neddylation). Numerous studies show that Cullin neddylation is essential for the activation of E3 ligase activity of CRL. De-neddylation, which removes the Nedd8 moiety, requires the isopeptidase activity of COP9 signalosome (CSN) that consist eight subunits in complex. De-neddylation results in the binding of inhibitory protein CAND1 to Cullin. Thus, neddylation stimulates the assembly of competent E3-substrate complexes with their cognate E2 enzymes and that de-neddylation facilitates the turnover of these complexes and results in the stabilisation of substrate proteins. It will be interesting to determine whether CSN2 expression correlates with tumourigenesis. Importantly, TNF-*α* also promotes the activation of the Wnt/*β*-catenin pathway through the suppression of GSK-3*β* activity in gastric tumour cells (Oguma *et al*, 2008). In addition, Wnt signalling leads to the sequestering of GSK-3*β* and the upregulation of Axin2 and thus induces EMT by inducing the stabilisation as well as nuclear localisation of Snail (Yook *et al*, 2006). Furthermore, Snail can enhance the activation of Wnt signalling by interacting with *β*-catenin and thus Snail establishes a positive feedback loop for Wnt-dependent transcription (Stemmer *et al*, 2008). Strikingly, both *β*-catenin and Snail are highly expressed in tumour cells at the invasive front (tumour-stromal boundary) in which the level of TNF-*α* is elevated. These cells lose the expression E-cadherin and they dissociate from the tumour mass and infiltrate into the surrounding stroma. It will be interesting to know whether the synergistic interaction of Snail and *β*-catenin is required for EMT induction and tumour cell invasion at the invasive front (Figure 2).

## Conclusion

Tumour necrosis factor-*α* clearly has a major role in establishing the link between inflammation and cancer. It contributes to the development of the tissue architecture necessary for tumour growth and metastasis. It also induces other cytokines, angiogenic factors and MMPs and thus contributes to the increased growth and survival of tumour cells. These tumour-promoting activities suggest that inhibition of TNF-*α* is an effective strategy for cancer therapy. Indeed, clinical trials with TNF-*α* antagonists are encouraging and show promising effects. For example, D2E7 (a fully humanised anti-TNF-*α* monoclonal antibody), infliximab (a chimeric immunoglobulin G1 monoclonal antibody against TNF-*α*), pegylated recombinant humanised sTNF-R1, pegylated humanised anti-TNF-*α* fragment (CDP870) and TNF-*α* synthesis inhibitors (p38 kinase inhibitors) have now been used to treat various tumours (Szlosarek and Balkwill, 2003; Garber, 2009). However, further investigation is required to determine whether these agents also inhibit Snail expression and suppress other EMT-associated signalling events.
